# Cyprus St. John’s Wort, *Hypericum repens* L.: Major Constituents, Antioxidant, Antimicrobial, and Anticholinesterase Activities

**DOI:** 10.3390/plants14121881

**Published:** 2025-06-19

**Authors:** Despina Charalambous, Eleni Kakouri, Antonis Ververis, Irene Zorba, Dionisis Chatzidakis, Marios Andreou, Kyproula Christodoulou, George A. Karikas, Petros A. Tarantilis

**Affiliations:** 1 Department of Pharmacy, School of Health Sciences, Frederick University, Nicosia 1036, Cyprusm.andreou@frederick.ac.cy (M.A.); hsc.kg@frederik.ac.cy (G.A.K.); 2 Laboratory of Chemistry, Department of Food Science & Human Nutrition, School of Food and Nutritional Sciences, Agricultural University of Athens, Iera Odos 75, 118 55 Athens, Greece; elenikakouri@aua.gr (E.K.); ptara@aua.gr (P.A.T.); 3 Neurogenetics Department, The Cyprus Institute of Neurology and Genetics, Nicosia 2371, Cyprus; antonisv@cing.ac.cy (A.V.); roula@cing.ac.cy (K.C.); 4 Nature Conservation Unit, Frederick University, Nicosia 1036, Cyprus

**Keywords:** *Hypericum repens* L., hydroethanolic extract, flavonoids, free radical scavenging activity, minimum inhibitory concentration

## Abstract

Chemical analysis of the hydroethanolic *Hypericum repens* L. extracts was performed using the LC/Q-TοF/HRMS technique. The majority of compounds identified belonged to phenolics, particularly flavonoids. The extract was also studied for its possible bioactivities, demonstrating high antioxidant properties compared to the control (IC_50_ values ranging from 4.6 to 9.42 μg/mL). Significant antibacterial activity was also detected against *Escherichia coli*, *Staphylococcus aureus*, *Enterococcus faecalis,* and *Salmonella enteritidis,* with MIC values from 125 to 625 μg/mL. *S. aureus* presented the highest susceptibility among all bacteria tested. Additionally, the extract showed very mild anticholinesterase activity in the AChE and BChE inhibition assays. These findings provide the first insights into the phytochemical composition, as well as the antioxidant, antimicrobial, and anticholinesterase activities of *H. repens* extract, suggesting that the endemic Cyprus *H. repens* is a valuable natural rich source of bioactive compounds with a potentially broad range of bioactivities.

## 1. Introduction

The genus *Hypericum*, belonging to the *Hypericaceae* family, encompasses approximately 500 diverse species of herbs, shrubs, and trees, flourishing predominantly in temperate climates and tropical mountainous regions. These plants are particularly abundant in Europe, Asia, Northern Africa, and North America, where they thrive in diverse ecological niches, showcasing their adaptability and resilience [1,2]. Throughout history, numerous *Hypericum* taxa have held significant traditional value as medicinal plants, revered for their efficacy in treating a broad spectrum of health conditions. The therapeutic applications of these species include addressing inflammation, combating bacterial and viral infections, facilitating the healing of burns, managing stomach ulcers, alleviating mild depression, and aiding in the treatment of diarrhea, pain, fevers, and even cases of poisoning from venomous animal bites [3,4]. *Hypericum perforatum* L. (St. John’s wort), the most popular species, is well known for its use in the treatment of mild to moderate depression, which makes it one of the best-selling dietary supplements. This wide acceptance in traditional medicine underscores the genus’s importance in folk remedies and modern therapeutic practices.

Beyond their traditional uses, *Hypericum* species are rich in various biologically active secondary metabolites, which exhibit an impressive spectrum of pharmacological effects. These effects encompass antimicrobial [5], anti-inflammatory [6], antioxidant [5], antifungal [7], astringent [4], antihyperglycemic [4], and hepatoprotective properties [4]. Furthermore, they demonstrate powerful inhibitory activities against critical enzymes, such as acetylcholinesterase and monoamine oxidase, highlighting their potential for treating various neurological disorders [5,8].

Prominent secondary metabolites isolated from the *Hypericum* genus include naphthodianthrones such as hypericin and pseudohypericin, known for their diverse therapeutic actions, and phloroglucinols, notably hyperforin, that have shown significant promise in managing mood disorders and other health issues [4]. A number of flavonoids, including quercetin, rutin, quercitrin, isoquercetin, hyperoside, and amentoflavone, have also been identified in *Hypericum* species, known for their antioxidant and anti-inflammatory properties [9,10].

The pharmacological attributes of *Hypericum* compounds extend to having demonstrated antifungal, antiviral, and anticancer properties, making them a pivotal focus of research in both pharmacognosy and medicinal chemistry [11,12,13,14]. As the exploration of these compounds continues, the potential for developing novel therapeutic agents from *Hypericum* species represents a promising frontier in modern medicine. Its botanical name derives from the Greek word hypericon (“υπέρ εικόνα”, meaning “above the icon”), suggesting its healing properties against the evil eye. *Hypericum* use has been reported even during classical antiquity by Hippocrates and Dioscorides [13].

**Figure 1:**
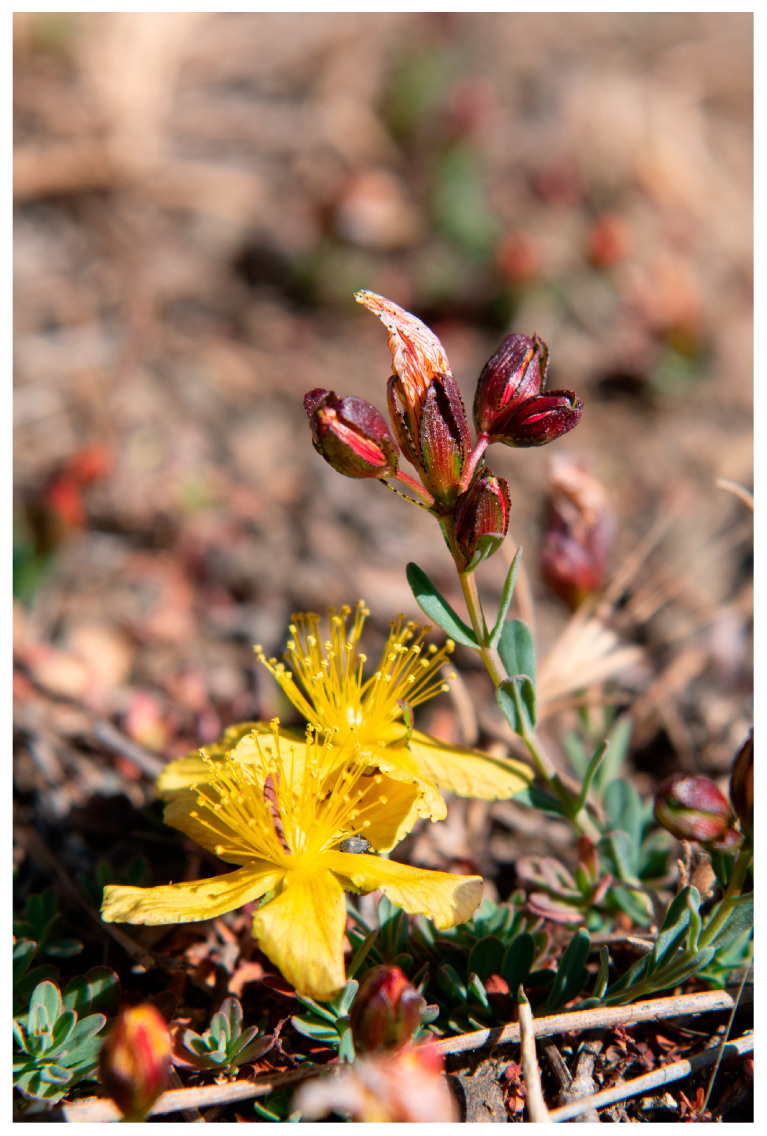
*Hypericum repens* L. flowers (© Marios Andreou).

*Hypericum repens* L. is a hemicryptophyte from the *Hypericaceae* family, growing up to 10 cm high. It is an endemic species of Cyprus, found in several areas of the island, i.e., Botanical Divisions 2 (Troodos), 3 (Limassol), 5 (Nicosia), and 7 (Kyrenia) [15,16]. It is a prostrate, perennial herb with rooting, hairless shoots up to 20 cm long. It has opposite leaves, simple, entire, elliptical to narrowly spathulate with a rounded top and black dots (glands) on the margin. Its flowers are actinomorphic, in cymose inflorescence, with golden yellow petals and red nerves (Figure 1). The plant blooms from May to July (August) and grows on dry, rocky slopes and openings in pine forests, wasteland, or field edges, on limestone or igneous formations at altitudes from 0 to 1450 m [15,16].

While the phytochemical profile of *H. perforatum* has been thoroughly investigated, several other *Hypericum* spp. still require further chemical analysis. To date, the chemical profile and biological properties of *H. repens* L. have not been evaluated. Thus, this work aimed to study the phytochemical content of *H. repens* L. hydroethanolic extract and assess its biological potential in terms of antioxidant, antibacterial, and cholinesterase inhibitory activity.

## 2. Results

### 2.1. Extraction Yield and Chemical Analysis of Hydroethanolic Extract of H. repens

Under the experimental conditions used in the present study, the extraction yield was calculated at 14.6 ± 1.1%.

Tentative identification of the compounds from *H. repens* extract was performed using LC/Q-TOF analysis. Results are presented in Table 1. Total ion chromatograms (TIC) and UV–Vis spectra are presented in the Supplementary Materials (Figure S1). According to our data, the extract is rich in flavonoids, mainly in their glycosylated form. In addition, procyanidin B-type dimers and procyanidin C1 (a B-type trimer) were detected, alongside quinic acid derivatives. Acylphloroglucinols and naphthodianthrones were not found in the sample.

### 2.2. Total Phenolic and Total Flavonoid Content

The total phenolic content (TPC) of the hydroethanolic extract of *H. repens* was evaluated using the Folin–Ciocalteu method. A standard curve of gallic acid was prepared, and the results were expressed as mg of gallic acid equivalents per gram of crude extract (mg GAE/g). The total flavonoid content (TFC) of the extract was estimated using the aluminum chloride colorimetric assay. A calibration curve was constructed using rutin, quercetin, and catechin reference standards and results were expressed as mg of rutin equivalents per gram of dry plant material (mg RUE/g), mg of quercetin equivalents per gram of dry plant material (mg QUE/g), and mg of catechin equivalents per gram of dry material (mg CAE/g). As presented in Table 2, the *H. repens* hydroethanolic extract demonstrated high TPC and TFC results, specifically for the rutin and catechin equivalents.

### 2.3. Antioxidant Activity

The antioxidant activity of *H. repens* hydroethanolic extract was examined using three different methods: two cell-free assays (DPPH and ABTS) and one cell-based assay (DCFDA). The *H. repens* extract demonstrated remarkable DPPH radical scavenging activities in vitro in a concentration-dependent manner (Table 3). The standard Trolox exhibited higher DPPH radical scavenging activity than that of the plant extract. The concentration of the studied plant extract required to scavenge 50% of the DPPH radicals (IC_50_) was also determined in this study. The IC_50_ value for the *H. repens* extract and the Trolox are presented in Table 4 (*p*-value < 0.01 **).

Regarding the antioxidant activity quantified by ABTS, the hydroethanolic extract also showed dose-dependent radical scavenging activity, although not as high as that of Trolox (Table 3). The IC_50_ values of the plant extract and the Trolox were also determined, and all the results are presented in Table 4 (values are significantly different, *p*-value < 0.01).

**Figure 2:**
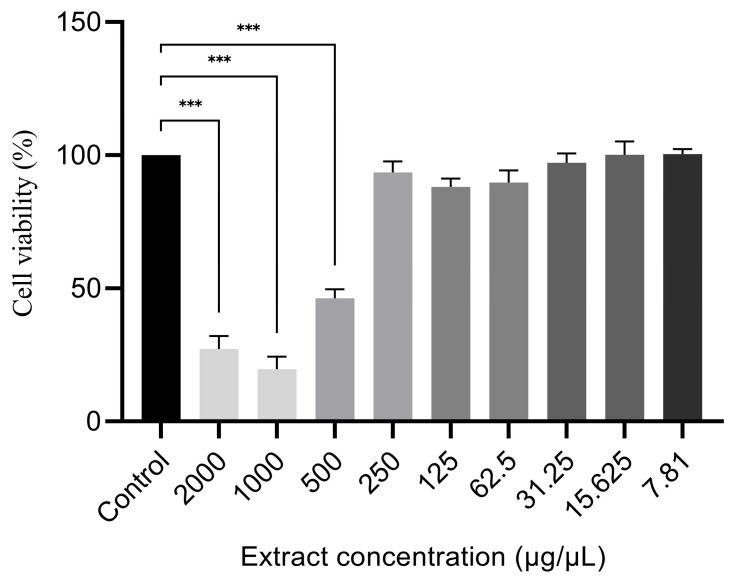
The effect of *Hypericum repens* L. hydroethanolic extract on SH-SY5Y cell viability was assessed using the MTT assay. *** indicates statistical significance at *p* < 0.001, compared to untreated control cells.

Before assessing the intracellular oxidative stress inhibition of the *H. repens* extract with the DCFDA assay, the extract was first tested for its cell toxicity using the MTT assay. The extract showed toxicity at concentrations of 500 μg/mL and above, indicating that higher doses may negatively impact cell survival or function. At these high concentrations, the extract most likely caused cellular stress or damage that led to reduced cell viability. However, no adverse effects were identified at concentrations below 500 μg/mL, suggesting that the extract may be safe for usage at lower levels (Figure 2). In the DCFDA assay, the extract demonstrated strong antioxidant activity, as evidenced by its IC_50_ value, which was comparable to the standard antioxidant Trolox (Table 4).

### 2.4. Antimicrobial Activity of Extracts

The minimum inhibitory concentration (MIC) of the hydroethanolic extract of *H. repens* was evaluated against Gram-negative (*E. coli*, *S. enteritidis*) and Gram-positive bacteria (*S. aureus*, *E. faecalis*). According to the results shown in Table 5, the plant extract demonstrated strong bacterial inhibition, with MIC values ranging from 125 to 312.5 μg/mL for all bacteria tested. Specifically, the inhibitory activity of the plant extract against *S. aureus* was higher compared to its activity against *E. coli*, *E. faecalis*, and *S. enteritidis*. Nonetheless, the antibacterial activity of the plant extract was weaker than that of ampicillin and gentamycin, as expected. Ampicillin and gentamycin were used as positive growth and sterility controls.

### 2.5. Anticholinesterase Assay

The acetylcholinesterase (AChE) and butyrylcholinesterase (BuChE) activity of *H. repens* was evaluated for the first time. All results are presented in Table 6. The extract exhibited very mild inhibitory activity against AChE and BuChE. The IC_50_ values for this activity were measured and are presented in Table 6. The synthetic drug donepezil hydrochloride, which is used to treat mild to moderate forms of Alzheimer’s dementia, was employed as the positive control. Its IC_50_ values against AChE and BuChE were also calculated (Table 6).

## 3. Discussion

The present study aimed to investigate the phytochemical composition of *H. repens* an endemic *Hypericum* species grown only in Cyprus. Furthermore, the hydroethanolic extract’s antioxidant, antimicrobial, and anticholinesterase activities were also documented.

Several chemical classes of secondary metabolites have been identified in *Hypericum* spp. extracts. Flavonoids are common to almost all the species studied so far, while naphthodianthrones and acylphloroglucinols are not consistently present [10,17]. *H. repen*s belongs to sect. *Oligostema*. According to Crockett et al., the characteristic compounds of this section include not only hypericin and pseudohypericin, but also other flavonoids such as quercetin, rutin, and I3-II8 biapigenin [18]. Plants of this section, such as *H. kelleri* Bald and *H. humifusu*m, have been previously evaluated for their phytochemical profile [19,20,21]. Compounds including quercetin, chlorogenic acid, hypericins, I3-II8 biapigenin, neo-chlorogenic acid, rutin, and quercitrin have been identified in the extracts. In our sample, despite the absence of naphthodianthrones and acylphloroglucinols, several other secondary metabolites were identified, apart from the majority of those mentioned before. B-type procyanidins and many flavonols were present, such as kaempferol and its glycosides, quercetin and its glycosides, hyperoside, and others. Quinic acid derivatives were also identified, along with mangiferin, a glucosylxanthone, frequently reported in other species of sect. *Oligostema* [22]. The data presented in this work contribute new insights into the chemical composition of the previously unexploited species *H. repens*, adding novel information concerning the phytochemistry of sect. *Oligostema.*

Recent studies have identified plants as significant sources of antioxidants, promoting their use in preventing and treating various health conditions [23]. Previously documented data on the antioxidant activity of ethanol extracts of *Hypericum perforatum*, the common *Hypericum* species, revealed considerable antioxidant potential, attributed to its phytochemical composition, including flavonoids, phenolic acids, and other bioactive compounds [24]. Additionally, other species (*H. tetrapterum*, *H. pubescens*, *H. montanum*) have been identified as potential sources of biologically active compounds and have shown a strong antioxidant/radical scavenging activity in both DPPH and ABTS assays [9,10]. Given the complex nature of oxidative processes and the varied characteristics of free radicals, it is crucial to employ a range of antioxidant assays to thoroughly assess the antioxidant properties of the extracts [25]. In combination with DPPH and ABTS methods, the DCFDA assay, an in vitro cell-based method, is also used to evaluate the efficacy of antioxidants present in plant extracts in scavenging intracellular reactive oxygen species (ROS) radicals. For these reasons, all three methods were employed to assess the antioxidant potential of the hydroethanolic *H. repens* extract. The extract demonstrated significant antioxidant activity, as evidenced by its IC_50_ value in all three methods. Comparatively, the IC_50_ value of the extract was close to that of the standard antioxidant Trolox. The latter highlights the strong antioxidant potential of *H. repens* and shows that the extract may serve as a valuable natural source of antioxidants, comparable to synthetic antioxidants like Trolox.

The potential of an extract to combat free radicals is certainly related to its phytochemistry. Beyond the occurrence of flavanones, flavanols, flavonols, and flavones, procyanidins are also reported as compounds with great antioxidant activity, attributed to their catechol group and their degree of hydroxylation [26,27]. In addition, the presence of biflavonoid amentoflavone and chlorogenic acid characterizes the extract’s overall antioxidant potency [8,28,29]. Generally, the major secondary metabolites found in the studied extract strongly support its dynamic role as an antioxidant agent.

*Hypericum perforatum* (St. John’s wort) is widely known for its use in treating mild to moderate depression, and there is also a growing body of evidence supporting its potential anticholinesterase activity. Altun et al**.** evaluated the anticholinesterase activity of *Hypericum perforatum* using both acetylcholinesterase and butyrylcholinesterase enzyme inhibition assays. The results indicated that the methanol extract of *Hypericum perforatum* demonstrated a potent inhibition of both acetylcholinesterase and butyrylcholinesterase, suggesting that it could potentially be used as a therapeutic agent in neurodegenerative conditions such as *Alzheimer’s* disease. The plant’s bioactive components, including hyperforin, flavonoids, and phenolic compounds, contribute to its potential as a natural agent for enhancing cognitive function [30].

Although several other *Hypericum* species have been tested for their anticholinesterase potential, there is no documented evidence for the anticholinesterase activity of *H. repens*. In the present study, the *H. repens* hydroethanolic extract exhibited very mild anticholinesterase activity in the AChE and BChE inhibition assays, indicating a relatively weak ability to interfere with these enzymes’ activity, when compared to the positive control donepezil, a well-known cholinesterase inhibitor employed in Alzheimer’s disease treatment [31]. The limited inhibition of AChE and BChE can be partly attributed to the flavonoids present in this plant. Thus, the different composition, either qualitatively or quantitatively, of the *H. repens* extract may account for the different AChE and BChE activity compared to other *Hypericum* species [32].

The anticholinesterase potential of the *H. repens* extract is comparatively lower than that observed in other *Hypericum* spp., such as *H. aciculare*, *H. hirsutum*, *H. rochelii*, *H. barbatum*, *H. tetrapterum*, *H. maculatum* ssp. Immaculatum, *H. perforatum*, *H. androsaemum*, and *H. undulatum*, and is similar to *H. triquetrifolium* [5,32,33,34]. In particular, a strongly lipophylic essential oil derived from *H. aciculare* wild plants from Ecuador exhibits stronger inhibition of AChE and BuChE than any other mixture obtained from a *Hypericum* species, with IC_50_ values close to that of donepezil [32]. Furthermore, the variation in bioactivity across different species from the same genus or plants from the same species originating from a different location demonstrates the importance of sourcing specific plant populations when targeting a specific biological property.

The current study has also shown that aerial parts of *H. repens* exhibit notable antibacterial activity against all four bacterial strains tested: *E. coli*, *S. aureus*, *E. faecalis*, and *S. enteritidis*. Previous research on nine *Hypericum* species grown in Greece has also reported antibacterial effects of hydroethanolic extracts against *S. aureus* and *E. faecalis*. Specifically, *H. perforatum, H. cycladicum*, *H. olympicum*, and *H. delphicum* showed strong selective antibacterial activity against Gram-positive bacteria [10]. Present data indicate a higher antibacterial activity compared to the antibacterial activity previously reported. Although IC_50_ values were higher than those calculated for the control compounds, namely ampicillin and gentamycin, the obtained results are encouraging, since *H. repens* extract inhibited the growth of both Gram(−) and Gram(+) bacteria. Its activity is partly attributed to the presence of procyanidins [35,36,37]. In this study, several types of procyanidin dimers and the procyanidin C1 trimer of epicatechin were detected. Data from the literature support that oligomeric procyanidins consisting of catechin–catechin or epicatechin–catechin dimers are the types of procyanidins to which strong antimicrobial activity is attributed [38,39]. Flavonoids, quinic acids, and the biflavonoid amentoflavone also contribute to the extract’s antimicrobial activity [39,40,41]. The structural diversity of flavonoids offers a variety of mechanisms for bacterial inhibition. Structure–activity relationship (SAR) studies of these compounds have been conducted in relation to their antimicrobial potential; however, the results are sometimes controversial. For example, Adamczak et al. and Cushnie et al. reported that the sugar moiety does not impede antimicrobial activity, whereas Bouarab-Chibane et al. stated the opposite [42,43,44]. Nevertheless, most studies agree that hydroxylation at positions 5C and 7C of the A ring for flavones, flavonols, and flavanols, and at position 3C for flavones, seems crucial for their activity [43,45,46]. In our study, most flavonoids detected were in their glycosylated form. In addition, flavones such as myricitrin and luteolin glycoside; flavanones such as astilbin and naringenin; and flavonols such as kaempferol and quercetin, fulfill SAR prerequisites regarding the position of hydroxyl groups. Generally, the presence of methoxy groups on ring C, the number and position of hydroxyl groups, the degree of polymerization, and the lipophilicity of the compound enhance or impede the interaction between flavonoids and bacteria [47]. Given their structural diversity, several mechanisms of action have been proposed [48,49], making flavonoids, and consequently, plant extracts, powerful antimicrobial agents.

## 4. Materials and Methods

### 4.1. Plant Material

Sampling was carried out with the coordinates of the central point of the surface being as follows: x: 34.902830; y: 32.924585, at an altitude of 1100 m. *H. repens* plants were identified and distinguished from other *Hypericum* species based on morphological characteristics, as previously described [14,15]. Species identification was carried out by Marios Andreou (co-author) and confirmed by Hadjikyriakou G. N. and Christodoulou C., experts in plant systematics and authors of the Flora of Cyprus website. The species was confirmed using samples CYP 656, 1409, 5331, 5913, 5916, and 5981, which are deposited in the Herbarium of the Department of Forests, Ministry of Agriculture, Rural Development, and Environment. Plant aerial parts (leaves and flowers) were collected during the flowering period from 100 randomly selected *H. repens* plants (total dry mass = 100 g) in the targeted area, without adversely affecting the population’s reproduction and survival. The total population size was approximately 300 plants.

### 4.2. Preparation of Extracts

*H repens* aerial parts were washed, air-dried at room temperature for 3–4 days, and crushed into fine powder. Four grams of dried plant material was extracted with n-hexane (CARLO ERBA reagents, Val de Reuil, France) to remove lipid compounds, in an ultrasonic water bath (GRANT type, Cambridge, UK) for 15 min at 35 kHz. Extraction was repeated in triplicate at 25 °C. The solid residue was dried under nitrogen steam and extracted with a hydroalcoholic solution (Fischer Scientific, Loughborough, Leicestershire, UK) (70% *v*/*v*) under the same experimental conditions. The extract was then placed on a rotary evaporator (Heidolph Laborota 4000 Rotary Evaporator) to remove the organic solvent, and the aqueous extract obtained was lyophilized (SP VirTis Freezemobile FM35EL−85–85 °C Freeze Dryer Lyophilizer). The collected powder, of deep purple color, was kept at −20 °C until further analysis. The percentage yield was calculated as follows:

% yield = [(weight of dry extract)/weight of dry material)] × 100 ± SD

### 4.3. Chemical Analysis of the Hydroethanolic Extract

The extract of *H. repens* was analyzed on an HPLC 1260 series system consisting of a degasser, quaternary pump, autosampler, diode array detector, and column oven (Agilent Technologies, Santa Clara, CA, USA). Separation of the compounds was performed on an EC100/4.6 Nucleoshell Bluebird RP-18, 2.7 μm, 100 mm × 4.6 mm column at 300 °C. The mobile phase consisted of H_2_O (A) (Genie A Ultrapure & RO Lab Water Systems, RephiLe Bioscience, Shanghai, China) and methanol (B) (LC/MS grade, Fischer Scientific, Loughborough, Leicestershire, UK), both acidified with 0.1% formic acid (99.0+%, Optima™ LC/MS Grade, Fisher Chemical™, Geel, Belgium). The elution conditions were as follows: flow rate 0.74 mL/min; column temperature 30 °C; injection volume 10 μL; detection wavelengths: 280, 300, 350, 550, and 590 nm. The following gradient program was applied: 0–0.5 min 25% B, 0.5–9 min 70% B, 9–15 min 90% B, 15–20 min 90% B, 20–21 min 25% B, 21–31 min 25% B.

The Q-TOF mass spectrometer was operated in both ESI(+) and ESI(−) ionization modes. The following parameters were set (equal for both ionization modes): capillary voltage 170 V, mass range 50–1700, gas temperature 350 °C, skimmer 65 V, octapole RF 750 V, drying gas 11 L/min, nebulizer pressure 450 psig, fragmentor voltage 170 V. Tandem mass spectra were recorded in the CID mode; m/z range was set to 40–900 and collision energy to 30 V.

A solution of *H. repens* (1.5 mg/mL) and standard solutions (each at a concentration of 0.5 mg/mL) of procyanidins B1 and B2, chlorogenic acid, epicatechin, quercitrin, kaempferol glucoside, quercetin, kaempferol, amentoflavone, and myricitrin (Extrasynthese, Genay Cedex, France) were prepared on the day of the analysis. Identification was based on the available standard solutions, observed *m*/*z* values of the ions, and the use of the “molecular feature extraction (MFE)” algorithm for unknown compounds.

Data were analyzed using the Agilent MassHunter Workstation software (ver. B. 07.00) for qualitative analysis.

### 4.4. Total Phenolic and Total Flavonoid Content Method

The total phenolic content (TPC) of *H. repens* hydroethanolic extract was determined using the Folin–Ciocalteu method, as previously described [50]. A standard gallic acid (Sigma Aldrich, Hamburg, Germany) curve (at dilutions of 0.025–1.5 mg/mL in methanol (Merck, Gillingham, UK)) was constructed. Absorbance was recorded at 750 nm after 30 min using a spectrophotometer (UV-1280, Shimadzu Europa GmbH, Duisburg, Germany). The same procedure was repeated with *H. repens* L. extract, prepared in dimethyl sulfoxide (DMSO, Sigma Aldrich, Hamburg, Germany).

The total flavonoid content of the extract was estimated using the aluminum chloride colorimetric assay. Crude extracts were dissolved in dimethyl sulfoxide (DMSO). Then, 250 μL of each extract was added to 1 mL of water and 75 μL of 0.7 M NaNO_2_ (Sigma Aldrich, Hamburg, Germany), and incubated for 5 min in the dark. Then, 75 μL 0.7 M AlCl_3_ (Sigma Aldrich, Hamburg, Germany) was added to the mixture and incubated for 15 min in the dark. Absorbance was read at 415 nm against a blank solution using the solvent. Calibration curves were prepared using rutin (Sigma Aldrich, Hamburg, Germany, at concentrations 0.02–0.2 mg/mL), quercetin (Sigma Aldrich, Hamburg, Germany, at concentrations 0.025–0.3 mg/mL), and catechin (Sigma Aldrich, Hamburg, Germany, at concentrations 0.02–0.5 mg/mL). Results were expressed as mg of standard equivalents per gram of dry plant material.

### 4.5. Cell Culture

SH-SY5Y human neuroblastoma cells were obtained from DSMZ (Braunschweig, Germany). The cells were regularly cultured in Dulbecco’s modified Eagle’s medium (Biosera, Cholet, France) supplemented with 10% fetal bovine serum (Capricorn Scientific, Ebsdorfergrund, Germany), 5% horse serum (Gibco, Grand Island, NY, USA), and 1% antibiotics (penicillin and streptomycin, Gibco, Grand Island, NY, USA), and maintained at 37 °C with 5% CO_2_.

### 4.6. Cell Viability Assay

The viability of SH-SY5Y cells was evaluated using the MTT assay following treatment with the *H. repens* extract [51]. Cells (2.5 × 10⁴ per well) were cultured in transparent 96-well plates and exposed to the extract for 48 h. Subsequently, 45 μg/mL of thiazolyl blue tetrazolium bromide (MTT, Sigma-Aldrich, Hamburg, Germany) was added to each well, and the plates were incubated for 4 h at 37 °C to allow for the formation of formazan crystals. After incubation, 150 μL of DMSO (Sigma Aldrich, Hamburg, Germany) was added to dissolve the crystals. The plates were then shaken for 30 min, and the absorbance was measured at 570 nm (Synergy H1 microplate reader, Agilent, Santa Clara, CA, USA).

Cell viability was determined by calculating the ratio of the absorbance of treated cells to that of control cells, with blank readings subtracted from all measurements. The assay was performed in triplicates in four independent experiments.

### 4.7. Antioxidant Activity of Hydroethanolic Extracts

#### 4.7.1. DPPH Method

The free radical scavenging activity of the hydroethanolic extract was assessed in vitro using the 2,2′-diphenyl-1-picrylhydrazyl (DPPH) assay, following previously described methods [52]. A stock solution of DPPH (0.5 mM in 100% ethanol; Sigma Aldrich, Hamburg, Germany) was prepared and 100 μL of this solution was added to different concentrations of the extract. The mixture was shaken thoroughly and incubated in the dark at room temperature for 30 min. The absorbance was then measured at 515 nm using a microplate reader (Sunrise, TECAN Trading LTD, Mannedorf, Switzerland). A control was prepared in the same way but without the sample. Trolox (Sigma Aldrich, Hamburg, Germany) standard solution was also diluted 1:2 and used as a reference sample. The scavenging activity was calculated based on the percentage of DPPH radical scavenged using the following equation:

(1) $$\%\;DPPH\;scavengin\;gactivity\;=\;\frac{\left(AB\;-\;AA\right)\;}{AB}\;\times \;100$$

where AB is the absorbance of the control sample and AA is the absorbance of the sample.

The half-maximal inhibitory concentration (IC_50_) was defined as the concentration of the extract (μg/mL) required to inhibit 50% of the DPPH radical.

#### 4.7.2. ABTS Method

ABTS (2,2′-azinobis (3-ethylbenzthiazoline-6-sulphonic acid)) cation scavenging activity was assessed as described in previous studies [53]. To prepare the ABTS radical cations, a 7 mM ABTS solution (Sigma Aldrich, Hamburg, Germany) was mixed with a 2.45 mM potassium persulfate solution (Sigma Aldrich, Hamburg, Germany) and left to react overnight in the dark, resulting in a dark-colored solution containing ABTS radical cations. Before use in the assay, the ABTS radical cation solution was diluted with 50% methanol (Sigma Aldrich, Hamburg, Germany) to achieve an initial absorbance of approximately 0.70 ± 0.02 at 734 nm. To measure the free radical scavenging activity, 100 μL of the extract was mixed with 1900 μL of the ABTS working solution. Trolox was also used as a reference sample. All samples were incubated for 30 min in the dark and the absorbance of each sample was recorded at 734 nm using a microplate reader (Sunrise, TECAN Trading LTD, Mannedorf, Switzerland). The percentage of inhibition of the ABTS radical cation was calculated using Equation (1) (Section 4.7.1). The antioxidant activity of test samples was expressed as IC_50_, the concentration necessary for a 50% reduction of ABTS.

#### 4.7.3. Assessment of Intracellular Oxidative Stress Inhibition (DCFDA Assay)

To assess the extracts’ potential to alleviate intracellular oxidative stress, SH-SY5Y cells (1.5 × 10^4^ cells/well) were plated in a black 96-well plate. After 24 h, the cells were treated for 45 min with 20 μM 2′,7′-dichlorodihydrofluorescein diacetate (DCF-DA, Sigma-Aldrich, Hamburg, Germany), a dye that becomes fluorescent upon oxidation by reactive oxygen species (ROS) [54]. Then, the cells were incubated with serial dilutions of the extract starting from 500 μg/mL, alongside 50 μM hydrogen peroxide (Merck, Darmstadt, Germany), for 1 h. The fluorescence intensities were assessed at Ex/Em = 485/535 nm in a microplate reader (Synergy H1, Agilent, Santa Clara, CA, USA). The blank readings were subtracted from all wells and the intensities were normalized to those of cells treated with hydrogen peroxide only. Trolox (Abcam, Cambridge, UK) was utilized as a standard antioxidant, and the assay was performed in triplicate in five independent experiments. SH-SY5Y human neuroblastoma cells were obtained from DSMZ (Braunschweig, Germany) and were cultured as described in Section 4.5.

### 4.8. Antibacterial Activity Method

The minimum inhibitory concentration (MIC) of hydroethanolic *H. repens* extract was determined using the broth microdilution method [55]. The extract was prepared as a 10 mg/mL starting solution in 10% DMSO and were subjected to 2-fold serial dilutions with Mueller Hinton Broth II (MHB, Liofilchem, Italy). Cultures of *E. coli* (NCTC 9001, Sigma Aldrich, Hamburg, Germany), *S. aureus* (NCTC 6571, Sigma Aldrich, Hamburg, Germany), *E. faecalis* (NCTC775, Sigma Aldrich, Hamburg, Germany), and *S. enteritidis* (WDCM 00030, Sigma Aldrich, Hamburg, Germany) were grown in Tryptic Soy Broth (TSB, Liofilchem, Italy) at a concentration of approximately 1 × 10^6^ cfu/mL using a spectrophotometer (V-630, Jasco International Co. LTD, Cremella, Italy). Blank samples (extract with no bacteria) were included as a control in TSB. Control wells contained bacteria but no extract to serve as growth controls, while sterility controls contained TSB with no bacteria or extract. Positive controls included wells with bacteria and either ampicillin (0.516 mg/mL, Sigma Aldrich, Hamburg, Germany) or gentamycin (0.064 mg/mL, Molekula, Darlington, UK). The MIC was determined after 18 h of incubation at 37 °C, followed by the addition of 30 μL of 0.2 mg/mL p-iodonitrotetrazolium chloride (INT, Sigma Aldrich, Gillingham, UK). After further incubation at 37 °C for 30 min, the absorbance at 492 nm was measured using a microplate reader (Sunrise, Tecan Trading Ltd., Mannedorf, Switzerland). The MIC was defined as the lowest concentration of the extract that completely inhibited bacterial growth, as indicated by the absence of color change in the medium, compared to the blank control.

### 4.9. Acetylcholinesterase (AChE) and Butyrylcholinesterase (BChE) Activity Inhibition Assay

AChE and BChE (Sigma Aldrich, Hamburg, Germany) were dissolved in 50 mM Tris-HCl buffer (pH 8) containing 0.1% (*w*/*v*) bovine serum albumin (Amersham Pharmacia Biotech, Amersham, UK) at a concentration of 5 U/mL. 5,5′-Dithiobis(2-nitrobenzoic acid) (DTNB, Sigma Aldrich, Hamburg, Germany) was dissolved in 50 mM Tris-HCl buffer (pH 8) supplemented with 0.1 M NaCl and 0.02 M MgCl_2_ at a 3 mM concentration. Acetylthiocholine iodide (ATCI, Sigma Aldrich, Hamburg, Germany) and S-butyrylthiocholine chloride (BTC, Santa Cruz, Heidelberg, Germany) were prepared in 50 mM Tris-HCl buffer (pH 8) at a concentration of 1.5 mM just before use. In each well of a transparent 96-well plate, 5 μL of extract dissolved in DMSO (Sigma Aldrich, Hamburg, Germany) was mixed with 88.5 μL of 50 mM Tris-HCl buffer (pH 8) and 1.5 μL of 5 U/mL AChE or BChE. In solvent control wells, the vehicle (DMSO) replaced the extract, while in blank control wells, 50 mM Tris-HCl buffer (pH 8) was used instead of all reagents. Finally, in the positive control wells, various concentrations of donepezil were added in place of the extract. The plate was shaken for 15 min at room temperature, and then 125 μL of 3 mM DTNB and 30 μL of 1.5 mM ATCI or BTC was added to each well. After shaking for 10 s, absorbance was measured at 405 nm using a Synergy H1 microplate reader at two time points: t = 0 and t = 30 min. Blank control readings were subtracted from all measurements, and the following formula was applied to calculate enzyme inhibition:

(2) $$\%\;AChE\;or\;BChE\;inhibitory\;activity\;=\;\frac{\left(\Delta A\;Solvent\;control\;-\;\Delta A\;Sample\right)}{\Delta A\;Solvent\;control}\;\times \;100$$

where ∆A represents the absorbance at t = 30 min minus the absorbance at t = 0.

The assays were performed in triplicate, with four independent experiments conducted.

### 4.10. Statistical Analysis

All experiments were performed in triplicate and the results were expressed as the mean value ± the estimated SD. Significance between the means was determined by Student’s t-test (*p* < 0.01). The statistical software GraphPad Prism version 8.0 was used for the statistical analysis.

## 5. Conclusions

In conclusion, the findings presented here contribute to a better understanding of the major chemical content and pharmacological properties of *H. repens*, highlighting its potential as an alternative medicine/nutritional supplement and a potent cosmetic constituent, due to its expected synergistic effects. Phytochemical analysis also documented abundance of flavonoids and phenolics (mainly procyanidin B-type dimers, procyanidin C1 (B-type trimer), and quinic acid derivates). Additionally, the study demonstrated the strong antioxidant activity of the extract. Quinic acid derivatives and procyanidins are well documented for their ability to neutralize free radicals and protect against oxidative stress. The study also demonstrated a significant antibacterial activity of the extract against Gram-positive and Gram-negative bacteria. Ongoing research aims to fully chemically characterize the bioactive compounds present in *H. repens*. Overall, the present experimental data offer valuable insights that could facilitate the utilization of *H. repens* L. in local and international pharmaceutical and cosmetic industries, toward a promising innovative product.

## Figures and Tables

**Table 1:** Tentatively identified compounds by LC-QTOF-MS/MS analysis of the aerial parts of the hydroethanolic extract of *Hypericum repens* L.

		ESI (+)				ESI (−)			
Tentatively identified compounds tR (min)	Molecular formula	*m*/*z* experimental	*m*/*z* theoretical	MS/MS product ions [M+H]^+^(relative abundance %)	Mass error Δm (ppm)	*m*/*z* experimental	*m*/*z* theoretical	MS/MS product ions [M−H]^−^(relative abundance %)	Mass error Δm (ppm)
Shikimic acid 1.42	C_7_H_10_O_5_	n.d. ^1^				173.0448	173.0455	111.0406 (1) 93.0372 (90) 87.009 (100) 44.9980 (6)	−4.04
Procyanidin C1 1.44	C_45_H_38_O_18_	867.2131	867.2131	715.1643 (10) ^2^ 577.1295 (23) 289.0676 (100) 127.0373 (52) 123.0478 (25)	0.00	n.d.^1^			
Cyanidin hexoside 1.45	C_21_H_20_O_11_	449.1076	449.1078	287.0543 (100)	−0.44	n.d.			
Procyanidin B1 2.12	C_30_H_26_O_12_	579.1442	579.1497	Standard solution ^3^	−0.34	n.d.			
Neo-chlorogenic acid 2.37	C_16_H_18_O_9_	n.d.				353.0873	353.0878	191.0555 (100) 179.0342 (1) 135.0447 (3)	−1.42
Procyanidin B2 3.23	C_30_H_26_O_12_	579.1497	579.1497	Standard solution	0.00	577.1347	577.1351	Standard solution	−0.69
Chlorogenic acid 3.89	C_16_H_18_O_9_	355.1019	355.1024	Standard solution	−1.41	353.0871	353.0878	Standard solution	−1.98
Epicatechin 4.18	C_15_H_14_O_5_	n.d.				289.0714	289.0717	Standard solution	−1.04
Phlorizin 4.2	C_21_H_24_O_10_	n.d.				435.1288	435.1296	271.0603 (100) 245.0823 (4) 167.0336 (2) 151.0393 (32) 125.0241 (47)	−1.84
Kaempferol dicoumaroyl hexoside 4.62	C_39_H_32_O_15_	741.1814	741.1814	323.0549 (4) 287.0524 (100) 163.0369 (21) 153.0161 (2) 123.0446 (7)	0.00	n.d.			
p-Coumaroylquinic acid 5.29	C_16_H_18_O_8_	n.d.				337.0917	337.093	191.0554 (100) 173.0464 (4) 163.0387 (2) 111.0458 (8)	−3.86
Mangiferin 5.27	C_19_H_18_O_11_	423.0937	423.0922	Standard solution		421.0764	421.0776	Standard solution	−2.85
Procyanidin B type (dimer isomer) 5.45	C_30_H_26_O_12_	579.1505	579.1497	425.0779 (4) 291.0911 (78) 287.0551 (100)	1.38	577.1338	577.1351	407.0769 (53) 289.0694 (100) 245.0794 (25) 205.0491 (10)	−2.25
Feruloylquinic acid 5.47	C_17_H_20_O_9_	n.d.				367.1019	367.1034	191.0552 (100) 173.0446 (6) 127.0380 (2) 93.0351 (41)	−4.09
Procyanidin B type (dimer isomer) 5.65	C_30_H_26_O_12_	579.1500	579.1497	578.33 (100) 291.0819 (11) 287.0551 (96)	0.5	n.d.			
Naringenin hexoside 5.58	C_21_H_22_O_10_	n.d.				433.114	433.114	313.0711 (100) 285.0604 (4) 271.0628 (15) 119.0487 (7) 93.0315 (4)	0.00
Procyanidin B type (dimer isomer) 5.88	C_30_H_26_O_12_	579.1500	579.1497	291.0817 (10) 287.0537 (100)	0.05	577.1336	577.1351	407.0761 (57) 289.0709 (100) 245.0807 (22) 205.0510 (7)	−2.6
Procyanidin B type (dimer isomer) 5.99	C_30_H_26_O_12_	n.d.				577.1340	577.1351	407.0757 (56) 289.0714 (100) 245.0806 (24) 205.0489 (6)	−1.9
Galangin hexoside 6.31	C_21_H_20_O_10_	n.d.				431.0974	431.0984	311.0552 (100) 269.0429 (3) 117.0337 (3) 105.0330 (1)	−2.32
Myricetin hexoside 6.50	C_21_H_20_O_13_	n.d.				479.0819	479.0831	316.0214 (100) 271.0229 (10) 151.0027 (2)	−2.5
Apigenin hexoside 6.55	C_21_H_20_O_10_	n.d.				431.0970	431.0984	311.0548 (100) 269.0445 (3) 161.0258 (1) 121.0287 (1) 117.0337 (4)	−3.25
Kaempferol rhamnoside 6.71	C_21_H_20_O_10_	433.1145	433.1129	287.0548 (100) 259.0554 (4) 153.0164 (4) 123.0446 (16)	1.38	n.d.			
Myricetin hexoside (isomer) 6.71	C_21_H_20_O_13_	n.d.				479.0816	479.0831	316.0213 (100) 271.0224 (10) 151.0016 (2)	−3.13
Astilbin 7.01	C_21_H_22_O_11_	n.d.				449.1079	449.1089	303.0502 (3) 151.0034 (100) 107.0232 (14)	−2.23
Taxifolin 7.16	C_15_H_12_O_7_	305.0652	305.056	287.0524 (2) 259.0578 (3) 195.0279 (4) 153.0171 (98) 149.0222 (100)	−1.31	n.d.			
Myricitrin 7.45	C_21_H_20_O_12_	465.1027	465.1027	Standard solution	0.00	463.0876	463.0882	Standard solution	−1.29
Epigallocatechin coumarate 7.47	C_24_H_20_O_9_	453.1190	453.1182	191.0330 (100) 165.0505 (10) 163.0376 (19) 137.0213 (37)	1.76	n.d.			
Kaempferol pentoside 7.53	C_20_H_18_O_10_	n.d.				417.0818	417.0827	327.0496 (100) 285.0387 (10) 241.0491 (1) 213.0543 (1) 199.0382 (1) 163.0027 (1)	−1.92
Hyperoside 7.72	C_21_H_20_O_12_	465.1022	465.1027	Standard solution	−1.07	463.0882	463.0876	standard solution	−1.29
Quercetin pentoside 7.87	C_20_H_18_O_11_	435.0925	435.0923	303.0493 (100) 165.0163 (1)	0.46	n.d.			
Isoquercitrin 7.94	C_21_H_20_O_12_	n.d.				463.0875	463.0882	300.0263 (100) 271.0251 (15)	−1.51
Quercetin pentoside (isomer) 7.95	C_20_H_18_O_11_	n.d.				433.0771	433.0776	301.0330 (63) 300.0265 (100) 283.0240 (2) 259.0605 (1) 152.0097 (2) 151.0029 (9)	1.15
Quercetin pentoside (isomer) 8.17	C_20_H_18_O_11_	n.d.				433.0782	433.0776	301.0336 (70) 300.0265 (100) 283.0243 (2) 259.0621 (1) 152.0106 (4) 151.0035 (11) 108.0214 (1)	1.38
Quercitrin 8.30	C_21_H_20_O_11_	449.1076	449.1078	Standard solution	−0.44	447.0925	447.0933	Standard solution	−1.79
Kaempferol glucoside 8.56	C_21_H_20_O_11_	n.d.				447.0923	447.0933	Standard solution	−2.24
Luteolin pentoside 9.24	C_20_H_18_O_10_	n.d.				417.0816	417.0827	327.05 (100) 285.0395 (20) 269.0420 (2) 241.0460 (2) 177.0151 (1) 133.0284 (7)	−2.64
Methylmyricetin 9.54	C_16_H_12_O_8_	n.d.				331.0451	331.0459	299.0196 (8) 271.0241 (100) 167.0313 (5) 151.0019 (68) 136.0151 (8)	−2.42
Quercetin 9.55	C_15_H_10_O_7_	n.d.				301.0350	301.0354	Standard solution	−1.33
Kaempferol 10.03	C_15_H_10_O_6_	n.d.				285.0401	285.0405	Standard solution	−1.4
I3-II8 biapigenin 10.92	C_30_H_18_O_10_	n.d.				537.0816	537.0827	267.0268 (1) 151.0035 (100) 117.0334 (1) 107.0137 (11)	−2.05
Amentoflavone 11.57	C_30_H_18_O_10_	539.0986	539.097	Standard solution	2.41	n.d.			

^1^ n.d.: not detected; ^2^ relative intensity of fragment ions; ^3^ identification of the compound was based on comparison of the retention time (tR) with a standard solution. Only MS analysis was performed on standard solutions.

**Table 2:** Total phenolic content and total flavonoid content of hydroethanolic *Hypericum repens* L. extract.

TPC	TFC		
mg GAE ^1^/g Crude Extract ± SD	mg RUE ^2^/g Crude Extract ± SD	mg QUE ^3^/g Crude Extract ± SD	mg CAE ^4^/g Crude Extract ± SD
24.920 ^a^ ± 0.023	12.872 ^b^ ± 4.33	4.844 ^c^ ± 0.475	12.289 ^b^ ± 1.045

Results are expressed as the mean values of three independent experiments. ^1^ mg GAE/g crude extract: mg gallic acid equivalents per gram of crude extract; ^2^ mg RUE/g crude extract: mg rutin equivalents per gram of crude extract; ^3^ mg QUE/g crude extract: mg quercetin equivalents per gram of crude extract; ^4^ mg CAE/g crude extract: mg catechin equivalents per gram of crude extract; SD: standard deviation. ^a–c^ Values with different letters differ significantly (*p* < 0.001).

**Table 3:** DPPH and ABTS scavenging activities of *Hypericum repens* L. hydroethanolic extracts.

Concentration (μg/mL)	DPPH Activity % Inhibition ± SD		Concentration (μg/mL)	ABTS Activity % Inhibition ± SD	
	Hydroethanolic	Trolox		Hydroethanolic	Trolox
15.63	77.69 ± 1.23	88.95 ± 3.45	12.5	84.86 ± 3.46	-
11.72	63.33 ± 0.98	85.33 ± 1.45	9.38	66.65 ± 1.37	90.43 ± 1.67
7.81	42.22 ± 0.78	81.88 ± 2.21	6.25	44.43 ± 1.23	86.98 ± 1.80
5.86	38.93 ± 0.34	69.90 ± 1.23	4.69	39.11 ± 0.96	69.65 ± 1.38
3.91	25.95 ± 2.34	46.60 ± 1.45	3.13	31.46 ± 1.64	46.43 ± 1.26
1.95	6.77 ± 0.67	23.65 ± 0.89	1.56	16.49 ± 0.52	22.65 ± 1.74
0.98	4.47 ± 0.43	14.72 ± 1.97	0.78	12.30 ± 1.73	13.77 ± 1.04
0.49	1.92 ± 0.31	15.89 ± 0.98	0.39	7.34 ± 0.62	7.09 ± 0.62
0.24	1.09 ± 0.21	11.87 ± 1.32	-	-	-
0.12	-	9.21 ± 0.34	-	-	-

Results are expressed as the mean values of three independent experiments. DPPH: 2,2-diphenyl-1-picrylhydrazyl; ABTS: 2,2′-azino-bis(3-ethylbenzothiazoline-6-sulfonic acid; SD: standard deviation.

**Table 4:** IC_50_ values for DPPH, ABTS, and DCFDA antioxidant activity of *Hypericum repens* L. hydroethanolic extracts.

Assay	IC_50_ Concentration (μg/mL) ± SD	
	Hydroethanolic	Trolox
**DPPH**	9.42 ± 0.56	4.44 ± 0.67
**ABTS**	6.78 ± 1.34	3.68 ± 0.73
**DCFDA**	4.68 ± 1.87	0.78 ± 0.14

Results are expressed as the mean values of three independent experiments. DPPH: 2,2-diphenyl-1-picrylhydrazyl; ABTS: 2,2′-azino-bis(3-ethylbenzothiazoline-6-sulfonic acid; DCFDA: 2′,7′-dichlorofluorescin diacetate; SD: standard deviation.

**Table 5:** Minimum inhibitory concentration (MIC) for *Hypericum repens L.* extract against *E. coli, S. aureus*, *E. faecalis*, and *S. enteritidis*.

MIC ^2^ (μg/mL)	*E. coli*	*S. aureus*	*E. faecalis*	*S. enteritidis*
*H. repens* L	312.5 ± 0.04	125 ± 0.07	312.5 ± 0.04	312.5 ± 0.09
**Amp ^1^**	16 ± 0.10	4 ± 0.15	8 ± 0.20	16 ± 0.12
**Gen ^1^**	2 ± 0.17	1 ± 0.23	16 ± 0.14	2 ± 0.25

^1^ Ampicillin and gentamycin were used as control antimicrobial agents. ^2^ The lower the MIC value, the less extract is needed for inhibiting the growth of the bacteria. Amp: ampicillin; Gen: gentamycin; MIC: minimum inhibitory concentration.

**Table 6:** IC_50_ values for Acetylcholinesterase and Butyrylcholinesterase inhibition by the *Hypericum repens* L. hydroethanolic extract.

Assay	IC_50_ Concentration (μg/mL) ± SD	
	Hydroethanolic	Donepezil
**AChE**	1317.69 ^a^ ± 40.69	0.04 ^b^ ± 0.01
**BuChE**	534.46 ^A^ ± 27.12	0.99 ^B^ ± 0.18

Results are expressed as the mean values of three independent experiments. AChE: acetylcholinesterase; BuChE: butyrylcholinesterase; SD: standard deviation. ^a,b,A,B^ Values with different letters differ significantly (*p* < 0.001).

## Data Availability

All data obtained and materials analyzed in this study are available from the corresponding author upon request.

## Supplementary Materials

The following supporting information can be downloaded at https://www.mdpi.com/article/10.3390/plants14121881/s1, Figure S1: Total ion chromatograms (TIC) and UV-Vis spectra of the hydroethanolic *H. repens* extract, generated by the LC/TOF/MS analysis at the negative ionization mode (A and A’) and at the positive ionization mode (B and B’).
