# Supplementary material for: Cyprus St. John’s Wort, Hypericum repens L.: Major Constituents, Antioxidant, Antimicrobial, and Anticholinesterase Activities
Source: Plants (Basel). 2025 Jun 19;14(12):1881. doi: 10.3390/plants14121881 (PMC12196913; doi:10.3390/plants14121881)
Supplement: Supplementary file 1 [file plants-14-01881-s001.zip › plants-3678801-supplementary.pdf]

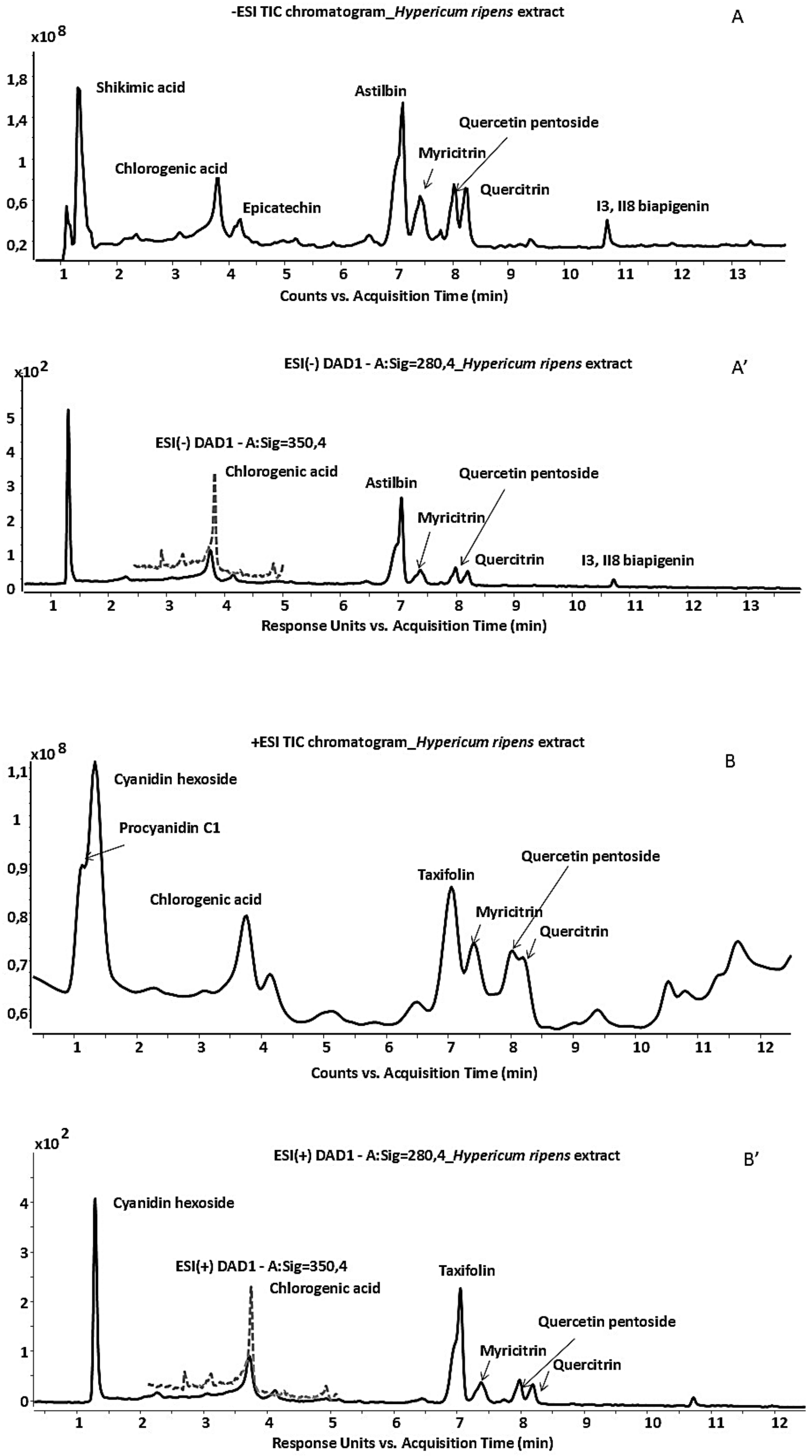

Figure S1: Total ion chromatograms (TIC) and UV-Vis spectra of the hydroethanolic extract *H. repens* L., generated by the LC/TOF/MS analysis at the negative ionization mode (A and A') and at the positive ionization mode (B and B').
